# Chronic *Mycobacterium avium* skin and soft tissue infection complicated with scalp osteomyelitis possibly secondary to anti-interferon-γ autoantibody formation

**DOI:** 10.1186/s12879-019-3771-3

**Published:** 2019-02-28

**Authors:** Xianli Xu, Xiaojie Lao, Chunlan Zhang, Cunwei Cao, Huarong Ding, Yu Pang, Qiuyue Ning, Jun Zou, Ning Zang, Diefei Hu, Maowei Chen

**Affiliations:** 1 Department of infectious disease, Guangxi Medical University First Affiliated Hospital, Nanning, China; 2 Department of dermatology and venereology, Guangxi Medical University First Affiliated Hospital, Nanning, China; 3 Department of burns and plastic surgery, Guangxi Medical University First Affiliated Hospital, Nanning, China; 4 Department of scientific education, The fourth People’s Hospital of Nanning, Nanning, China; 5 Medical Scientific Research Center of Life Sciences Institute, Guangxi Medical University, Nanning, China; 6 Department of infectious disease, Wuming Hospital of Guangxi Medical University, Nanning, China

**Keywords:** *Mycobacterium avium*, Infection, Skin

## Abstract

**Background:**

Nontuberculous mycobacterial (NTM) disease is commonly an opportunistic infection frequently found in immunocompromised individuals, but sometimes can also be found in the immunocompetent hosts, especially in East Asians. The NTM separation rate in China is increasing, which reminds us to focus on NTM infections in immunocompromised populations.

**Case presentation:**

A 43-year-old woman with a recurrent fever for more than 8-month and a right forehead surgical wounds unhealed for more than 6-month was admitted to our hospital on February 22, 2018. On arrival, several elliptic ulcers were obvious on the right forehead with pus and fibrin exudation, and the skin around the lesions was tender, reddish, no sense of fluctuation. The result of HIV serology test was negative. CD4+ T cell count was normal and tuberculosis antibody was negative. CT of the chest and head showed bone destruction. Skin biopsy on the right forehead was performed on March 13, 2018, and pathological examination of the excisional biopsy specimen found inflammatory granuloma and suppurative inflammatory changes. Broad-spectrum antibiotics were treated but the effect seemed discontent. Then debridement and skin grafting were performed on the right frontal ulcer under general anesthesia on April 3, 2018. The skin tissue culture that resected on March 13, 2018 found Nontuberculous mycobacteria grown after 78 days, so clarithromycin, ethambutol, protionamide, and amoxicillin clavulanate potassium were prescribed for anti-nontuberculous mycobacteria treatment beginning on May 31, 2018. In reviewing the case, *Mycobacterium avium* (*M. avium*) was identified in the skin tissue resected on April 3, 2018 by polymerase chain reaction (PCR) and the serum test of anti-interferon-γ autoantibodies was positive.

**Conclusions:**

This is a case report of “*Mycobacterium avium* SSTI (skin and soft tissue infection) and OM (osteomyelitis) with possible secondary immunodeficiency syndrome induced by anti-interferon-γ autoantibody”.

## Background

NTM is a mycobacterium different from the *Mycobacterium tuberculosis* complex and M. leprae [1]. NTM are environmental opportunistic pathogens of humans and animals [2, 3], which are widely found in human habitats, including drinking water distribution systems and household water and plumbing [3]. NTM can cause a wide array of clinical diseases; the pulmonary disease is most frequent, followed by lymphadenitis in children, skin disease, and other extrapulmonary or disseminated infections in severely immunocompromised patients [4], such as malignancy or infection with human immunodeficiency virus (HIV) [5]. Sometimes NTM infection can also be found in the immunocompetent hosts, especially in East Asians [6].

MAC was one of the most identifiable mycobacterial, which often lead to disseminated infections. A study found it had an extremely high prevalence rate (97.8%) of IFN-γ autoantibodies in patients with disseminated NTM infection [5].

Here is a case of *Mycobacterium avium* infection with skin symptom as the initial manifestation which doesn’t have a predisposing condition or immunosuppression, but the testing result of IFN-γ autoantibodies was positive. We aim to We aim to raise awareness of NTM infections when a patient presents with unexplained rashes, poor efficacy of medical therapy and surgery, and IFN-γ autoantibodies–associated immunodeficiency should be considered when NTM infection happens to immunocompetent hosts.

## Case presentation

**Fig. 1:**
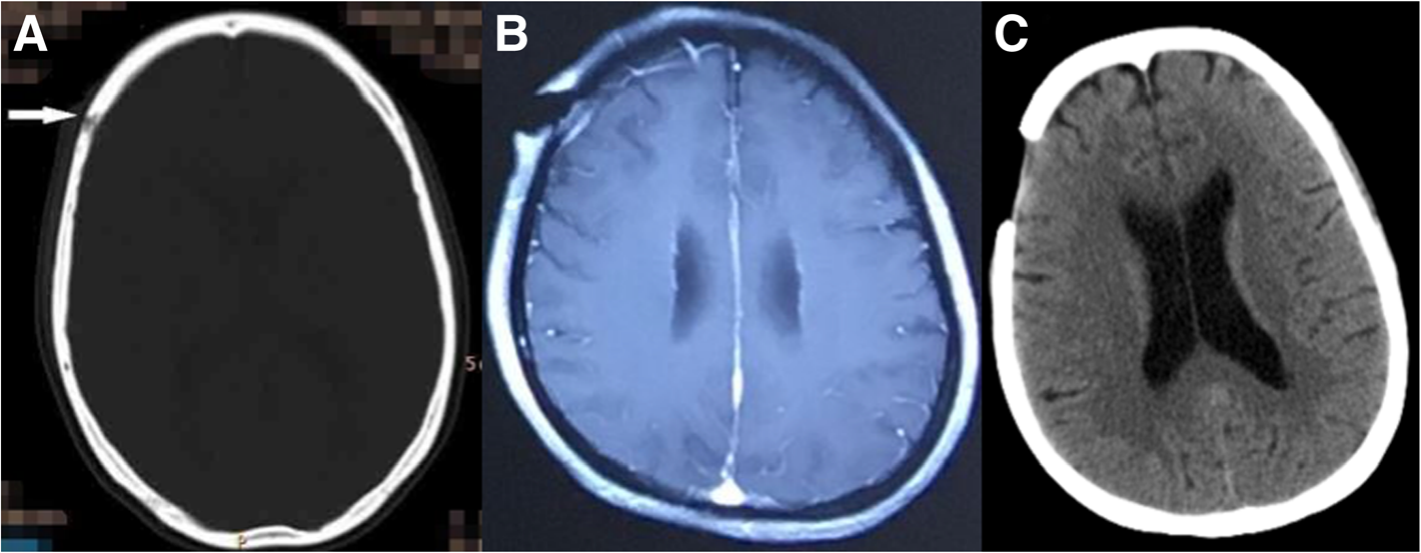
**a-c** Are computed tomography sequences of the head. **a** was taken in August 2017, which showed the skull damaged (arrow). **b** was taken in September 2017, which showed bone loss and destruction at the corresponding place. **c** was taken in February 2018, showing a postoperative change of the right frontal

A 43-year-old woman was admitted to our hospital with a recurrent fever for more than 8-month and a right forehead wound disunion after the mass excision for more than 6-month. In June 2017, she incidentally found a bean-size lump over the right forehead that was gradually increasing in size. Her symptoms were accompanied with recurrent fever but body temperature was not measured. In August 2017, the patient underwent surgical removal of the mass in a local hospital. The postoperative histopathology of the mass showing fibers and granulation tissue formation, accompanied with a high number of lymphocytes, plasma cells, neutrophils infiltration, and tiny abscess formation. After the operation, the patient had pain and swelling at the wound site along with a discharge of pus. Computed tomography (CT) of the head showed bone loss and destruction at the corresponding place (Fig. 1a). In September 2017, she underwent a debridement on the infected scalp and destructive bone of the right forehead (Fig. 1b). The pathology showed acute suppurative osteomyelitis with a high number of inflammatory hyperplasia, pus formation, and massive bone necrosis. The pathology of the right forehead mass revealed bleeding, purulent inflammatory changes, epidermis necrosis, and negative staining with PAS. The patient was impaired wound healing, and the wound oozing. Over the next few days, the patient has high fever which usually persists unremittingly (up to 40 °C), and the patient may continue to have recurrent rigors. Broad-spectrum antibiotics treatment seemed not effective. Then she was admitted to our department in February 2018. The patient reported no significant past medical history, and she denied any exposure to contaminants or suspected water sources.

**Fig. 2:**
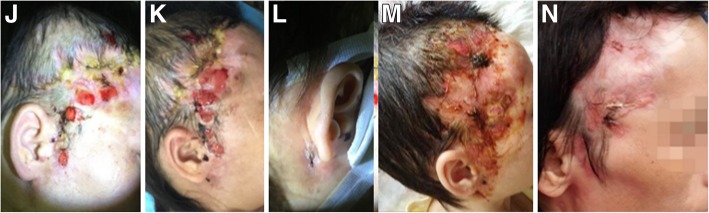
**j-n** Are pictures of the patient’s right frontal facial lesions. **j** showed the patient’s initial lesions. **k** and **l** were taken on March 19, 2018, which showed a newly appearing nodule behind the right ear accompanied with redness and ulceration. **m** was taken 1 month after anti-NTM treatment, showing the skin lesions almost healed. **n** was taken on 4 months after anti-NTM treatment, showing the skin lesions healed

Physical examination showed low body temperature (35.7 °C), several elliptic ulcers on the right forehead with pus and fibrin exudation (2.0 cm × 1.0 cm). The skin around the lesions was tender, reddish, no sense of fluctuation (Fig. 2j). There also had bilateral cervical lymphadenopathy (0.7 cm × 0.8 cm). Respiratory sounds and cardiac and abdominal examination were normal.

**Fig. 3:**
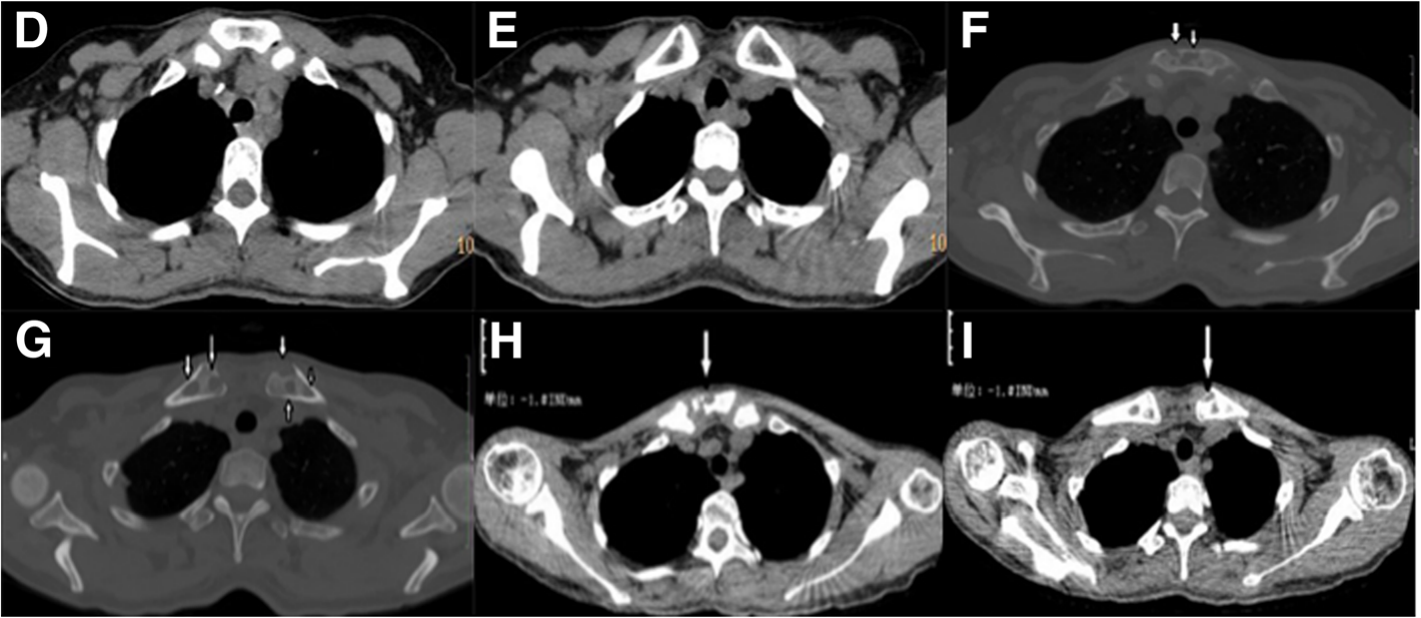
**d-i** Are chest computed tomography sequences. **d** and **e** were taken in August 2017, which showed no visible bone destruction in the sternum. **f** and **g** were taken in February 2018, which showed the bones of sternal stem and bilateral clavicle sternum stalk destructed. **h** and **i** were taken in June 2018, showing bone destruction of sternal stem and clavicle sternal stem

**Fig. 4:**
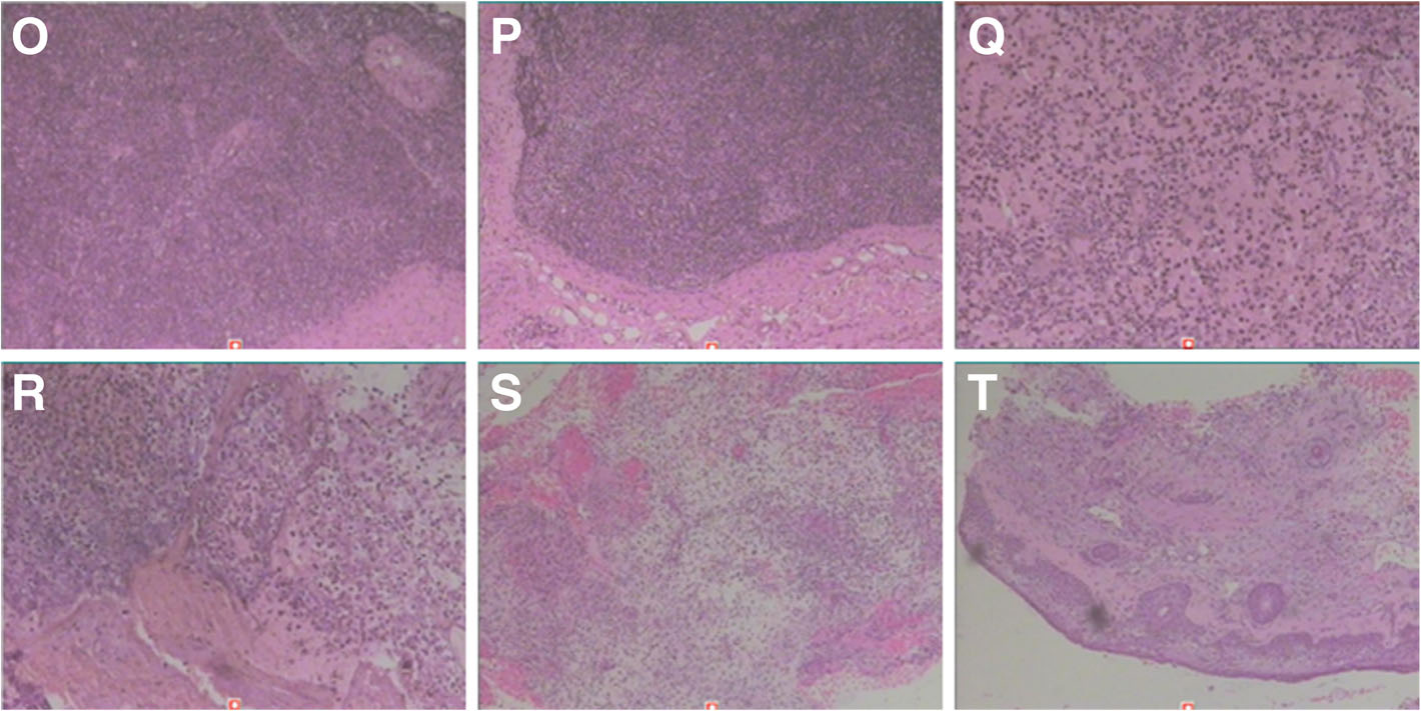
**o-t** Are pictures of pathological change. **o** and **p** showed lymph node reactive hyperplasia. **q** and **r** showed the pathological changes of the right frontal temporal bone with acute suppurative osteomyelitis and the skin tissue with inflammatory granulation tissue hyperplasia in September 2017. **s** and **t** showed the pathological changes of the skin tissue resected on March 13, 2018 with inflammatory granulation

Laboratory testing revealed a leukocyte count of 17.13 × 10^9^/L with 82.1% of neutrophils, a C-reactive protein concentration of 192.00 mg/L, a procalcitonin of 0.247 ng/ml and a negative result of HIV serology test. CD4+ T cell count was normal and the levels of serum globulins including IgG, IgA, IgM and total IgE were within the normal reference range. Tuberculosis antibody was negative, Hemoglobin 71.40 g/L and serum albumin 25.0 g/L. Other laboratory results were unremarkable. Ultrasonography revealed bilateral cervical, bilateral axillary and bilateral inguinal lymphadenopathy. CT of the chest revealed mild pneumonia, nodular opacity in the left upper lobe, a minimal pleural effusion on the left, and mildly enlarged lymph nodes in the mediastinal and in bilateral axillary (Fig. 3f, g). CT of the head showed the right frontotemporal soft tissue mildly swelling (Fig. 1c). The pathology of the cervical lymph node biopsy showed reactive hyperplasia (Fig. 4o, p). A bone marrow aspiration was done, and the cytology suggested mature neutrophils mostly with reactive changes with the neutrophil alkaline phosphatase (NAP) was 365. Lumbar puncture (LBP) was carried out. We found that the CSF pressure was normal and the spinal fluid examinations showed no abnormality. Skin biopsy on the right forehead was performed on March 13, 2018, and pathological examination of the excisional biopsy specimen found nothing but inflammatory granuloma and suppurative inflammation changes, with PAS and acid-fast staining negative (Fig. 4s, t). Blood, bone marrow, spinal fluid, wound secretion, skin and soft tissue Specimens cultures were all negative for bacteria, tuberculosis, and fungus. Because Mycobacterium culture technique was limited, the skin tissue which excised on March 13, 2018 was sent to another hospital for mycobacterial culture and antimicrobial susceptibility test. The diagnosis was as follows: 1. Right frontotemporal skin and subcutaneous tissue infection; 2. Infectious fever. She received broad-spectrum antibiotics treatment such as Piperacillin Sodium and Tazobactam Sodium combined with amikacin, linezolid with imipenem, moxifloxacin with teicoplanin and voriconazole, levofloxacin with clindamycin and itraconazole. Concomitantly the patient was treated with strengthening the dressing, increasing circulation, enhancing immunity and other supportive therapy.

However, a new tender and reddish tubercle occurred behind the right ear, then the tubercle enlarged and eventually broke down to form an ulcer (Fig. 2k, l). On April 3, 2018, debridement and skin grafting were performed on the right frontal ulcer under general anesthesia. Postoperative anti-infective treatment and dressing changing were continued. The temperature gradually decreased but still had a low-grade temperature (the highest daily temperature was around 37.6 °C), and part of the head skin graft survived with residual wound base granulation growth. The patient was discharged from our hospital on April 14, 2018.

On May 30, 2018, the mycobacterial culture and antimicrobial susceptibility test result returned which showed that Nontuberculous mycobacteria growth was noted after 78 days of culture in a Mycobacterium Growth Indicator Tube, sensitive to ethambutol and protionamide, resistant to streptomycin, isoniazid, rifampicin, aminosalicylic acid, levofloxacin, capreomycin, and amikacin. The patient was admitted again on May 31, 2018. The test of *Mycobacterium tuberculosis* complex DNA of her phlegm liquid was negative. Chest CT scan showed a few infected foci in the right middle lobe, irregular bone destruction of manubrium sternum and the sternal end of the bilateral clavicle, and the bone changes of corpus sternum and cervical vertebra (Fig. 3h, i). The infection caused by nontuberculous mycobacteria was confirmed, and clarithromycin, ethambutol, protionamide, and amoxicillin clavulanate potassium were prescribed for anti-nontuberculous mycobacteria treatment. There was no abnormality when monitoring blood routine and serum chemistry, and the effusion of wound gradually reduced. The patient was discharged on July 14, 2018, and she was reviewing regularly. We are still treating the patient with anti-nontuberculous mycobacteria regimen above-mentioned and change the dressing regularly.

## Discussion and conclusions

An epidemiological survey showed that the rate of NTM strain isolation was up to 22.9% in 2010 in China [7]. The clinical manifestations of NTM diseases include respiratory tract infections, disseminated infections, skin and soft tissue infections, lymphadenitis, ocular infections, and so on [8].

Skin and soft tissue infections (SSTIs) caused by NTM are underrecognized, due to their wide spectrum of clinical presentations and histopathological findings that are often nonspecific [9, 10]. The manifestation of skin disease presents with nodules, subcutaneous abscesses, pustules, ulcers, or combinations thereof [4]. The patient in our case was presented with the manifestation of nodules, ulcers.

Multi-disciplinary collaboration is necessary for diagnosis, including the clinician, the histopathologist, the microbiologist, and infectious disease specialists [4, 10]. In this case, the patient developed a right forehead mass with fever, and the wound did not heal after the mass resection. The broad-spectrum antibiotic treatment was not effective. Histopathological findings showed purulent inflammation with infiltration of lymphocytes and neutrophils. Finally, the right frontal skin biopsy tissue was cultured nontuberculous mycobacteria, so the diagnosis of the nontuberculous mycobacterial disease was established. Culture is still the gold standard, because of limited sensitivity and specificity of symptoms, radiology, and direct microscopy of clinical samples [4].

Disseminated NTM diseases present as two distinct clinical syndromes [4]. The patient’s head CT showed bone destruction in the corresponding site of the lesion. Chest CT scan showed irregular bone destruction in the sternum stem and bilateral clavicle sternum, bone changes in the sternum and cervical vertebrae. Thus NTM bone disease was not excluded. The corresponding bone tissue NTM cultural examination is required. This patient could have disseminated NTM disease, but the diagnosis of disseminated NTM disease requires blood or bone marrow culture positive. Currently, positive evidence for blood or bone marrow culture was absent.

**Fig. 5:**
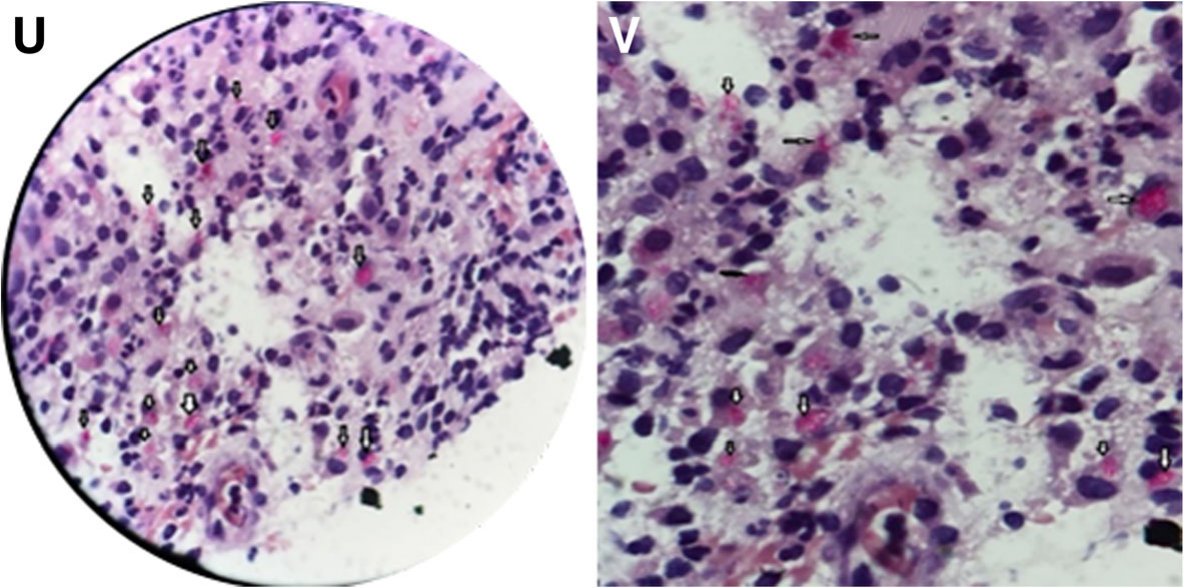
**u-v** Are the picture of acid-fast staining (the second time)

A variety of modalities including tissue culture and polymerase chain reaction (PCR) assays are crucial in order to identify the organism [10]. Reviewing the case, we re-stained the skin tissue which was excised on April 3, 2018 with acid-fast staining, and the results showed positive (see Fig. 5). Then the *Mycobacterium avium* (*M. avium*) was identified by PCR. Owing to the uncertainty of manual operation and sampling, we consider that acid-fast staining of the tissue slices was related to the content of tissue samples and the thickness of slices, so there is a certain rate of missed diagnosis.

*M. avium* is one of the important pathogens in disseminated disease, whereas M. intracellular is one of the common respiratory pathogen [11]. The disseminated NTM disease mainly occurred in immunocompromised patients, especially in HIV-infected patients. The patient in our case was previously healthy and HIV negative and her CD4/CD8 lymphocyte count was normal. However, we tested the patient for IFN-γ autoantibodies and the result was positive.

Neutralizing anti–interferon-γ autoantibodies were detected in 88% of Asian adults with multiple opportunistic infections and were associated with an adult-onset immunodeficiency similar to advanced HIV infection [12]. High titers of IFN-γ autoantibodies are mainly found in patients with the disseminated non-tuberculous mycobacterial disease and are more common in East Asian women who have not been treated with exogenous interferon gamma [13]. IFN-γ autoantibodies are evidence of acquired immunodeficiency that should be examined in cases of unexplained disseminated NTM infections in Asian-born persons [14]. In our case, the patient developed an NTM infection without a history of immunodeficiency and other chronic diseases, but IFN-γ autoantibodies proved to be positive. Hence acquired immunodeficiency should be considered, such as adult-onset immunodeficiency syndrome.

Treatment can be challenging, as it can be dependent on multiple factors, including the causative organism, the patient’s immunological status, and the extent of disease involvement [10]. The optimal regimen against disseminated MAC is clarithromycin, ethambutol, +/−rifabutin, whether a three-drug regimen alone in this setting would be adequate is not known. The optimal duration of treatment is also unknown, but 6 to 12 months of chemotherapy is usually recommended [11, 15]. Specific treatment for IFN-γ autoantibodies associated NTM infection is not codified and required prolonged, multiple-drug regimens. Using immunomodulation strategies is still debated, and long-term suppressive treatment should be taken into account for persisting high levels of neutralizing antibodies [14].

According to the culture results and drug susceptibility test, clarithromycin, ethambutol, protionamide, amoxicillin-clavulanate potassium regimen were given to treat NTM infection for our patient. One month later, the patient’s right forehead wound gradually healed and there was no new rash.

In summary, chronic *Mycobacterium avium* skin and soft tissue infection complicated with scalp osteomyelitis possibly secondary to anti-interferon-γ autoantibody formation. This case underscores the need for clinicians to be aware of the potential for NTM infection when a patient presenting with unexplained rashes, poor efficacy to medical therapy and surgery. When NTM infection is detected in an immunocompetent patient, IFN-γ autoantibodies–associated immunodeficiency should be considered.

## Abbreviations

CSF: Cerebrospinal fluid
CT: Computed tomography
DNA: Deoxyribonucleic acid
HIV: Human immunodeficiency virus
IFN-γ: Interferon gamma
IgA: Immunoglobulin A
IgE: Immunoglobulin E
IgG: Immunoglobulin G
IgM: Immunoglobulin M
IHC: immunohistochemistry
LBP: Lumbar puncture
*M. avium*: *Mycobacterium avium*
MAC: *Mycobacterium avium*- intracellular complex
NAP: Neutrophil alkaline phosphatase
NTM: Nontuberculous mycobacterial
PAS: Periodic Acid-Schiff stain
PCR: Polymerase chain reaction
SSTIs: Skin and soft tissue infections
